# Pathogenesis and Phylogenetic Analyses of Two Avian Influenza H7N1 Viruses Isolated from Wild Birds

**DOI:** 10.3389/fmicb.2016.01066

**Published:** 2016-07-07

**Authors:** Hongmei Jin, Deli Wang, Jing Sun, Yanfang Cui, Guang Chen, Xiaolin Zhang, Jiajie Zhang, Xiang Li, Hongliang Chai, Yuwei Gao, Yanbing Li, Yuping Hua

**Affiliations:** ^1^College of Wildlife Resources, Northeast Forestry UniversityHarbin, China; ^2^Harbin Veterinary Research Institute, Chinese Academy of Agriculture SciencesHarbin, China; ^3^Research Institute of Forestry Ecology, Environment and ProtectionBeijing, China; ^4^Hubei Province Wildlife Epidemic Disease CenterWuhan, China; ^5^Changchun Veterinary Research Institute, Chinese Academy of Agricultural SciencesChangchun, China

**Keywords:** H7N1, H7N9, Avian influenza virus, phylogenetic analysis, pathogenic analyses

## Abstract

The emergence of human infections with a novel H7N9 influenza strain has raised global concerns about a potential human pandemic. To further understand the character of other influenza viruses of the H7 subtype, we selected two H7N1 avian influenza viruses (AIVs) isolated from wild birds during routine surveillance in China: A/Baer's Pochard/Hunan/414/2010 (BP/HuN/414/10) (H7N1) and A/Common Pochard/Xianghai/420/2010 (CP/XH/420/10) (H7N1). To better understand the molecular characteristics of these two isolated H7N1 viruses, we sequenced and phylogenetically analyzed their entire genomes. The results showed that the two H7N1 strains belonged to a Eurasian branch, originating from a common ancestor. Phylogenetic analysis of their hemagglutinin (HA) genes showed that BP/HuN/414/10 and CP/XH/420/10 have a more distant genetic relationship with A/Shanghai/13/2013 (H7N9), with similarities of 91.6 and 91.4%, respectively. To assess the replication and pathogenicity of these viruses in different hosts, they were inoculated in chickens, ducks and mice. Although, both CP/XH/420/10 and BP/HuN/414/10 can infect chickens, ducks and mice, they exhibited different replication capacities in these animals. The results of this study demonstrated that two low pathogenic avian influenza (LPAI) H7N1 viruses of the Eurasian branch could infect mammals and may even have the potential to infect humans. Therefore, it is important to monitor H7 viruses in both domestic and wild birds.

## Introduction

Currently, avian influenza outbreaks and epidemics, particularly those of the H5 or H7 subtype, result in huge economic losses to the poultry industry and pose a serious threat to human health (Senne et al., 1996). Over the past two decades, many infections with influenza virus subtype H7 have occurred. For example, between 1999 and 2000, a H7N1 virus outbreak in Italy resulted in the death of more than 13 million chickens and caused extensive economic losses (Capua et al., 2003). In 2003, 30 million birds were culled in the Netherlands, Belgium, and Germany after an H7N7 subtype influenza outbreak. During that outbreak, the H7N7 subtype avian influenza virus (AIV) also infected 89 people, causing conjunctivitis with one fatal case (Fouchier et al., 2004). In February 2013, human infections with a novel H7N9 AIV were first reported in China and caused a widespread public health concern. By the end of March 2016, the H7N9 virus had infected 619 people and caused 255 fatalities in China (http://www.nhfpc.gov.cn/zwgkzt/ptggg/list.shtml). In addition, the emergence of the novel human H7N9 LPAI viruses in 2013 (Yu et al., 2015) demonstrated that H7 LPAI viruses can infect humans. However, wild aquatic birds, particularly waterfowl, waders and gulls, are regarded as a major natural reservoir of LPAI viruses, and these birds generally remain healthy while carrying the viruses (Alexander, 2000). Therefore, strengthening AIV surveillance in water birds is required.

In this study, we isolated a strain of H7N1 AIV from a healthy Baer's Pochard during AIV surveillance in Hunan Province in 2010. The same year, we also isolated a strain of H7N1 AIV from a healthy Common Pochard in Jilin Province. To better understand the molecular and biological properties of the two strains, we performed phylogenetic analysis based on complete genomic sequence data and assessed the replication and pathogenic potential of the two isolated viruses in ducks, chickens and mice. We also analyzed the receptor binding characteristics of the two H7 isolates. These studies expand our understanding of the latent evolutionary and transmission features of the H7 subtype viruses and would aid in disease control and pandemic preparedness efforts.

## Materials and methods

### Ethics statements

All animal studies were approved by the Institutional Animal Care and Use Committee of the Harbin Veterinary Research Institute, Chinese Academy of Agricultural Sciences. All animal procedures were carried out in strict accordance with the recommendations in the Guide for the Care and Use of Laboratory Animals of the Ministry of Science and Technology of the People's Republic of China. All experiments were performed in a biosafety level 2+ laboratory (enhanced animal biosafety level 2 laboratory and a negative pressure-ventilation laboratory) at Harbin Veterinary Research Institute (Harbin, China).

### Viruses used in the study

In our study, the avian influenza virus strain, A/chicken/Jilin/HU/02 (H5N1), which can specifically bind to α-2,3-linked sialic acid receptors, and the human influenza virus strain A/Jilin/31/2005 (H1N1), which can specifically bind to α-2,6-linked sialic acid receptors, were used. The strains were stored at Changchun Veterinary Research Institute, Chinese Academy of Agricultural Sciences. A/Baer's Pochard/Hunan/414/2010 (BP/HuN/414/10) (H7N1) and A/Common Pochard/Xianghai/420/2010 (CP/XH/420/10) (H7N1) isolated from a Baer's Pochard in Hunan Province and a Common Pochard in Jilin Province, respectively, were used in this study.

### Virus isolation

The two viruses analyzed in this study, BP/HuN/414/10 (H7N1) and CP/XH/420/10 (H7N1) were isolated during avian influenza surveillance in 2010. The method used to isolate these viruses involved oropharyngeal and cloacal swabs or fecal samples being suspended in antibiotic (1000 ug/ml penicillin and streptomycin)-treated phosphate-buffered saline (PBS) and centrifuged at 5000 rpm for 10 min at 4°C. The allantoic cavities of 10-day-old embryonated specific-pathogen free (SPF) chicken eggs were inoculated with the supernatant of oropharyngeal and cloacal swabs or fecal samples. The presence of AIV was confirmed with RT-PCR (See Supplementary Table 2). The 50% egg infection dose (EID_50_) was calculated according to the method described by Reed and Muench (1938). The allantoic fluid of the purified virus was stored at −80°C until use. All procedures were performed under aseptic conditions.

### Genome sequencing and phylogenetic analysis

Viral RNA was extracted from the allantoic fluid using TRIZOL Reagent (Invitrogen Carlsbad, CA, USA) and reverse transcribed using the primer 5′-AGCRAAAGCAGG-3′. The PCR products of eight fragments of the H7N1 virus were sequenced with a set of specific sequencing primers. The sequence data were compiled with the SEQMAN program (DNASTAR, Madison, WI). All reference sequences used in this study were obtained from the National Center for Biotechnology Information (NCBI) GenBank database and the Global Initiative on Sharing All Influenza Data (See Supplementary Table 1). DNASTAR's MegAlign was used to perform the sequence homology and key amino acid site analyses. MEGA 6.06 software was used to perform multiple sequence alignments with the Clustal W algorithm, and a phylogenetic tree was generated with the neighbor-joining method and bootstrap test (1000 replicates) based on the sequences for the open reading frames. Potential glycosylation sites were analyzed with the NetNGlyc 1.0 online software (www.cbs.dtu.dk/services/NetNGlyc/). GenBank accession numbers are JQ973643-JQ973650 for BP/HuN/414/10 and KU663402-KU663409 for CP/XH/420/10.

### Infection of chickens and ducks

To examine the replication and transmission of the two isolated H7N1 viruses in chickens and ducks, two groups of 4-week-old SPF chickens (white leghorn) and 3-week-old SPF ducks (shelduck) (eight birds/group) were inoculated with 100 μl 10^6^ EID_50_ virus in each bird. Twenty-four hours after inoculation, three additional chickens and ducks were placed in the same isolation units to monitor contact infection (Fan et al., 2015). All birds were monitored every day for clinical symptoms or death until 21 days post infection (dpi). Viral shedding was monitored through sampling of oropharyngeal and cloacal from infected and contacted birds at 3, 5, and 7 dpi. At 3 dpi, three birds in each group were euthanized, and tissues, including the brain, kidney, spleen, lung, bursa, trachea, cecal tonsil, thymus, and pancreas, were collected aseptically for virus titration. Serum samples were collected from each bird at 14 and 21 dpi for detection of antibodies using the HI assay.

### Infection of mice

To investigate the virulence of the two H7N1 viruses in mice, two groups (86-week-old female BALB/c mice/group) were lightly anesthetized with CO_2_ and inoculated intranasally with 50 μl of 10^6^ EID_50_ of the H7N1 influenza virus (Chen et al., 2004). At 3 dpi, three inoculated mice were euthanized with a peritoneal injection of sodium pentobarbital at a dose of 200 mg/kg, and their organs, including the lung, kidney, spleen, turbinate, and brain, were collected for viral titration and histopathological evaluation. The titers for virus infectivity in eggs were calculated with the method described by Reed and Muench (1938) and Li et al. (2005). The remaining five mice in each group were monitored daily for 14 days for weight loss and mortality (Zhao et al., 2012). The control group (five mice) was mock infected with PBS and monitored daily for 14 days for weight loss and mortality. All weight changes were calculated based on the average of five mice using the percent of the everyday weight divided by the first-day weight and multiplied by 100 (Ye et al., 2016). Significant changes in body weight were calculated by one-way ANOVA, and *P* < 0.05 was considered statistically significant (Mancinelli et al., 2016).

### Analysis of receptor specificity of the two strains of H7N1 virus

To prepare the red blood cell suspension, Alsever's solution anticoagulant was added at a dilution of 1:1 upon collection of chicken and sheep red blood cells. The chicken and sheep red blood cells were washed three times with PBS, centrifuged at 2000 rpm for 5 min at 4°C each time, and adjusted to final working concentrations (10 and 1%, respectively) with PBS and stored at 4°C.

For the sialidase treatment, 90 μl of a 10% suspension of chicken red blood cells was treated with 10 μl of α-2,3-sialidase (50 mU/μl) (TaKaRa, Dalian, China) for 10 min at 37°C. The sample was then washed two times with PBS, centrifuged at 2000 rpm for 5 min at 4°C each time, adjusted to a final working concentration (0.75%) with PBS, and stored at 4°C. The chicken red blood cells were treated with α-2,3-sialidase to eliminate all receptors except for the α-2,6-linked sialic acid receptor.

For the vibrio cholera neuraminidase (VCNA) (TaKaRa, Dalian, China) treatment, 90 μl of a 10% suspension of chicken red blood cells was treated with 10 μl of VCNA (50 mU/μl) for 1 h at 37°C, washed three times with PBS, centrifuged at 2000 rpm for 5 min at 4°C each time, adjusted to a final working concentration (0.75%) with PBS, and stored at 4°C. The chicken red blood cells were treated with VCNA to eliminate the α-2,3- linked sialic acid receptors and α-2,6-linked sialic acid receptors.

Both the 10% chicken red blood cells (with α-2,3-linked sialic acid receptors and α-2,6-linked sialic acid receptors) and 1% sheep red blood cells (with only α-2,3-linked sialic acid receptors) were then diluted to a concentration of 0.75%. Four virus suspensions, including BP/HuN/414/10, CP/XH/420/10, A/Jilin/31/2005 (H1N1), and A/chicken/Jilin/HU/02 (H5N1) were subsequently diluted with PBS to a dilution of 1:32, and the agglutination of red blood cells caused by diluted BP/HuN/414/10, CP/XH/420/10, A/Jilin/31/2005 (H1N1), and A/chicken/Jilin/HU/02 (H5N1) was determined using sheep red blood cells (0.75%), chicken red blood cells (0.75%), chicken red blood cells treated with a-2,3-sialidase (0.75%), and chicken red blood cells treated with VCNA (0.75%), respectively (Sun et al., 2008). This experiment was completed at the Changchun Veterinary Research Institute, Chinese Academy of Agricultural Sciences.

## Results

### Mutation analysis

Q226L and G228S mutations were not detected in the hemagglutinin (HA) protein, which indicates that the two H7N1 viruses may retain the characteristic of preferential binding to avian-like α-2,3-linked sialic acid receptors (Stevens et al., 2006; Yamada et al., 2006). Q226L and G228S mutations in the HA protein were not detected in the A/Shanghai/13/2013 (H7N9) strain, whereas HA S138A and T160A mutations were found in the two H7N1 viruses as well as the A/Shanghai/13/2013 (H7N9) strain. The two isolated viruses showed no E627K and D701N mutations in the PB2 protein, which plays an important role in the adaptation of AIVs to mammals (Katz et al., 2000; Li et al., 2005), but the E627K mutation was detected in the A/Shanghai/13/2013 (H7N9) strain. The amino acid substitution S31N was not detected in the M2 protein, indicating that these viral strains are sensitive to amantadine inhibitors (Lee et al., 2008), but it was detected in the A/Shanghai/13/2013 (H7N9) strain. The two H7N1 viruses and A/Shanghai/13/2013 (H7N9) exhibited mutations at position P42S of the NS1 protein (Table 1), which can increase virulence in mice (Jiao et al., 2008).

**Table 1:** **Selected characteristic amino acids of H7N1 subtype AIVs isolated from wild birds**.

**Gene**	**Key residues**	**Comments**	**The isolates**		
			**CP/XH/420/10 (H7N1)**	**BP/HuN/414/10 (H7N1)**	**A/Shanghai/13/2013 (H7N9)**
HA (H3 numbering)	Cleavage sites	Characteristic of low-pathogenic in chickens (Steinhauer, 1999)	PELPKGR↓GLFGAI	PELPKGR↓GLFGAI	PEIPKGR↓GLFGAI
	Ser138Ala(S-A)	Favor mammalian adaptation (Fan et al., 2015)	A	A	A
	Thr160Ala(T-A)	Increased affinity toward α-2,6-linked sialic acid receptor (Fan et al., 2015)	A	A	A
	Gln226Leu(Q-L)	Increased affinity toward α-2,6-linked sialic acid receptor (Yamada et al., 2006)	Q	Q	I
	Gly228Ser(G-S)	Increased affinity toward α-2,6-linked sialic acid receptor (Yamada et al., 2006)	G	G	G
PB2	Glu627Lys(E-K)	Mammalian adaptation (Li et al., 2005)	E	E	K
	Asp701Asn(D-N)	Mammalian adaptation (Li et al., 2005)	D	D	D
M2	Ser31Asn(S-N)	Amantadine resistance (Lee et al., 2008)	S	S	N
NS1	Pro42Ser(P-S)	Increased virulence in mice (Jiao et al., 2008)	S	S	S

### Phylogenetic analysis

To clarify the genetic relationship of the two H7N1 viruses, we sequenced the entire genome of each virus and compared the eight gene segments of each virus with sequences of typical influenza viruses obtained from the NCBI GenBank database (https://www.ncbi.nlm.nih.gov/genbank/) and Global Initiative on Sharing All Influenza Data (http://platform.gisaid.org).

**Figure 1:**
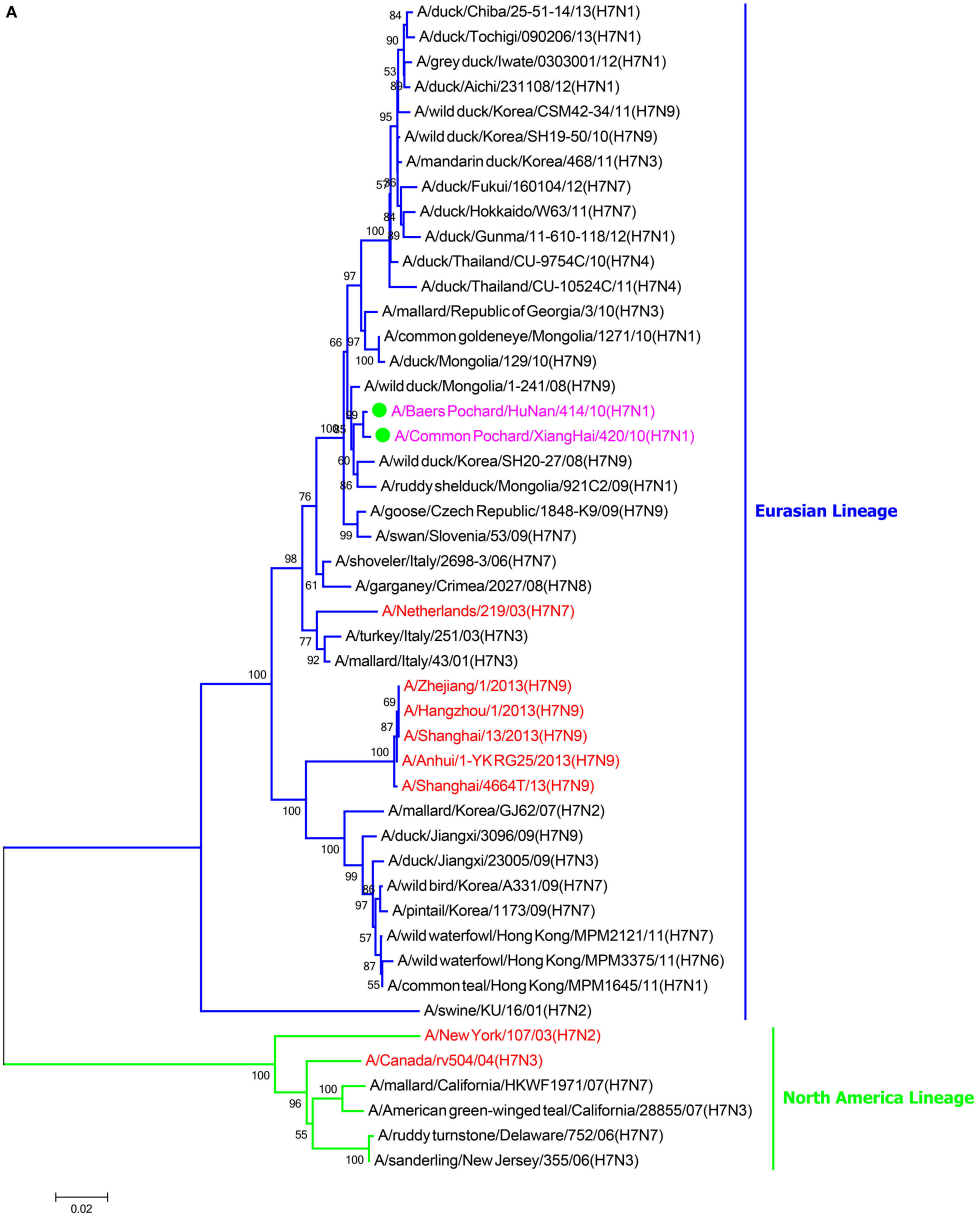

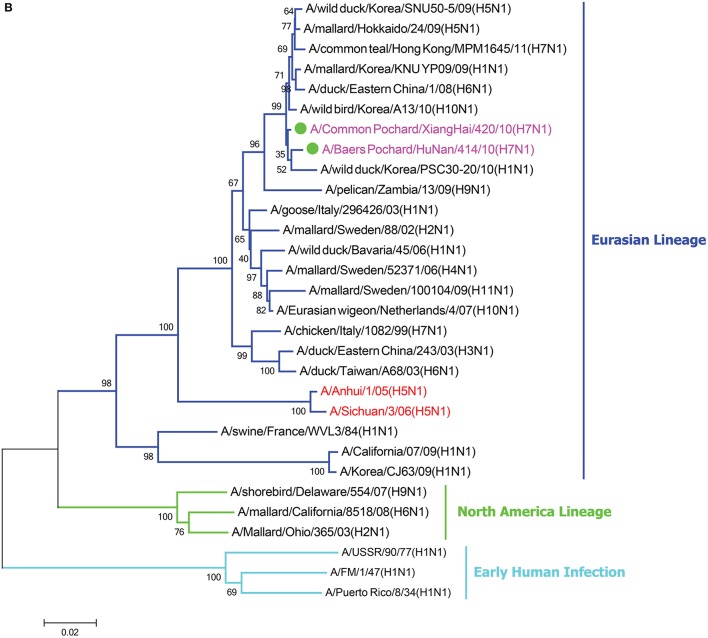

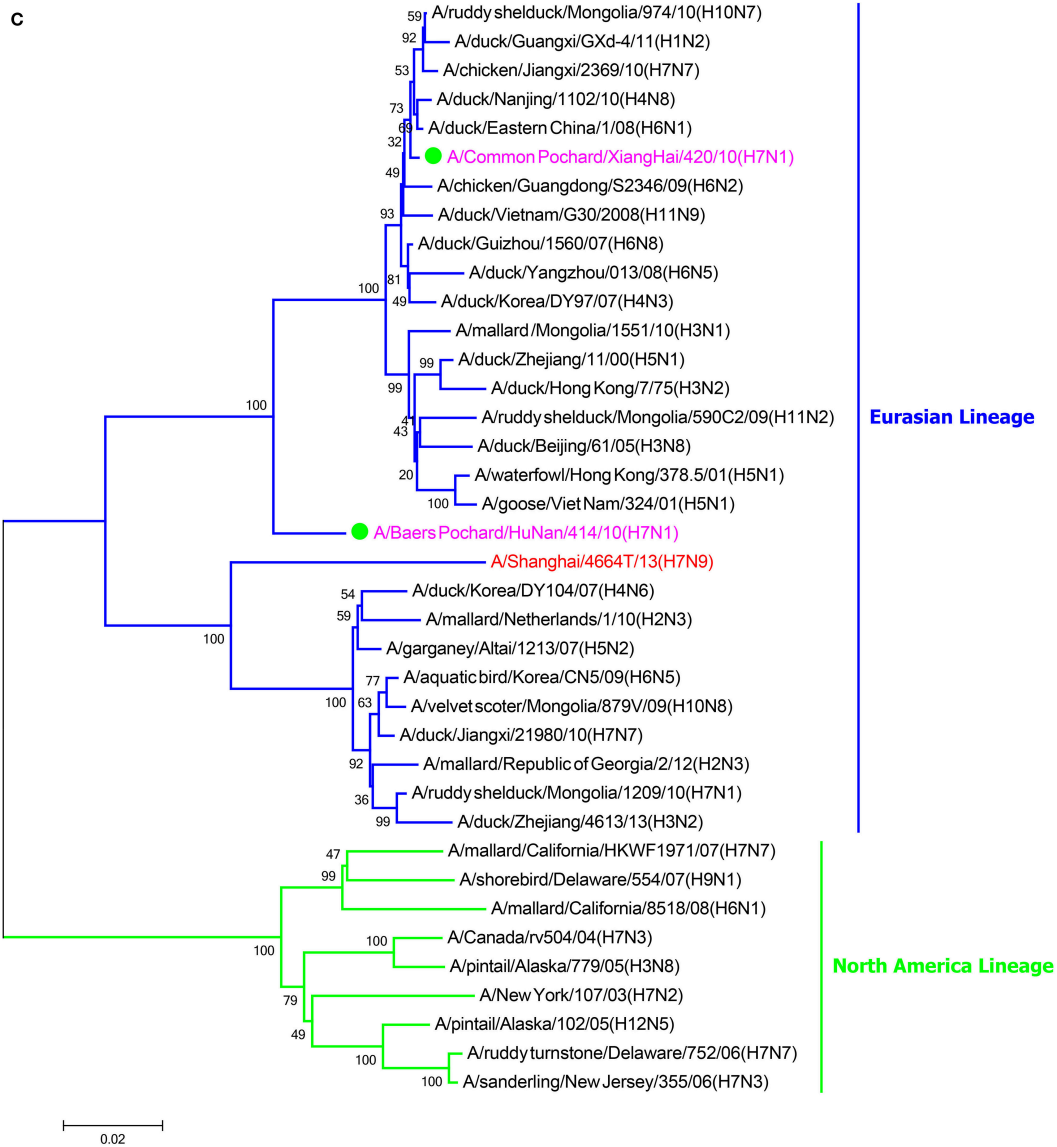

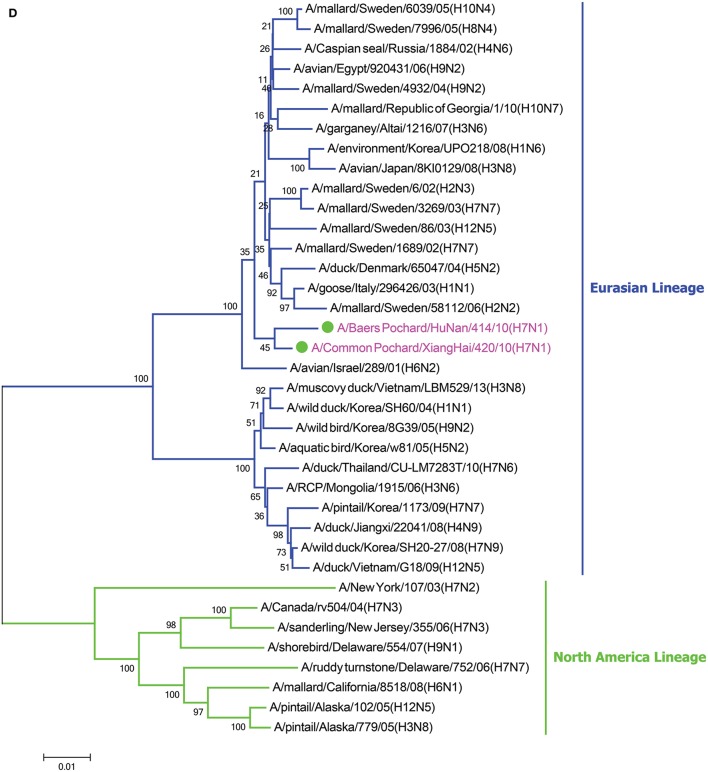

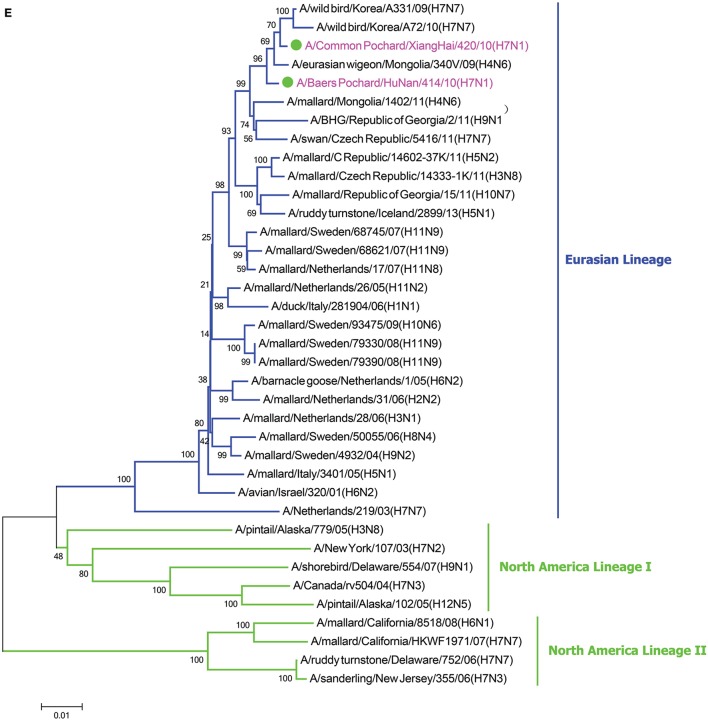

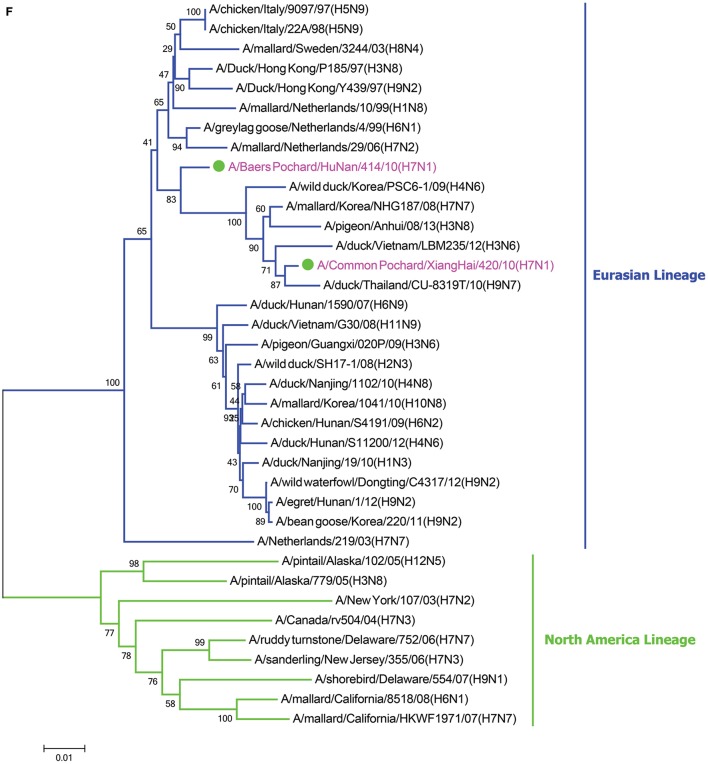

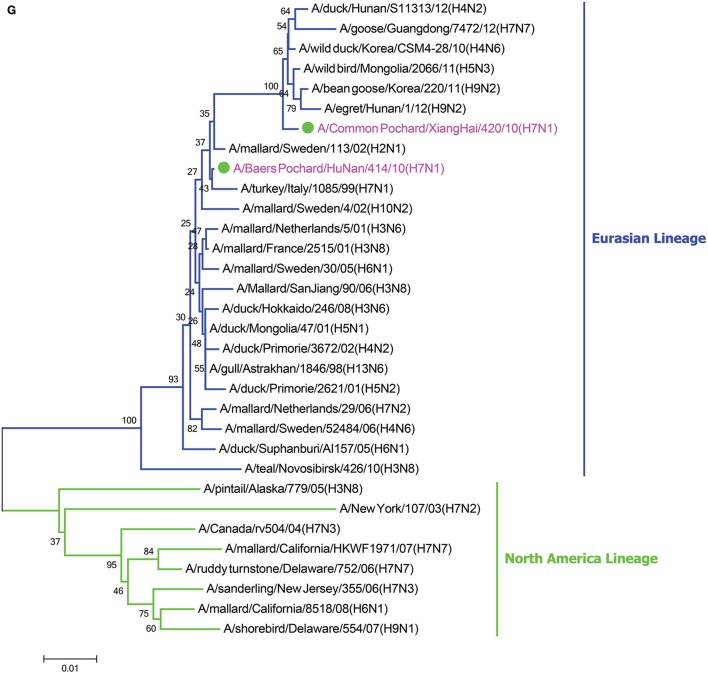

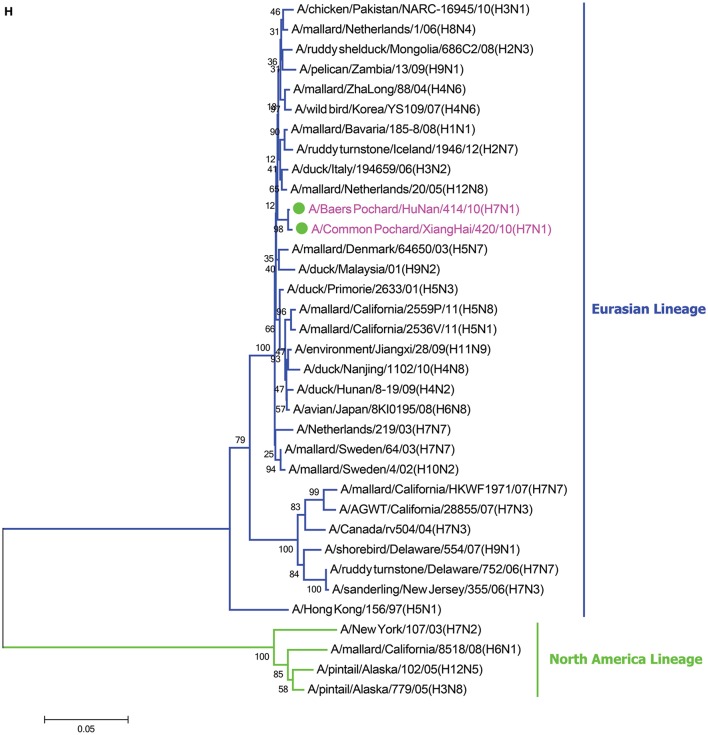
**A phylogenetic tree based on the open reading frame sequences of the HA (A), NA (B), PB2 (C), PB1 (D), PA (E), NP (F), M (G), and NS (H) gene segments of two H7N1 AIVs**. The two isolated H7N1 viruses are indicated by a closed circle and highlighted in purple. Human isolates are highlighted in red. Different lineages are highlighted in different colors. The trees were constructed using the neighbor-joining method of MEGA6.0 6 with 1000 bootstrap trials to assign confidence to the groupings.

In the HA phylogenetic tree (Figure 1A), the two viruses clustered into the Eurasian branch, and both viruses shared a close genetic relationship with 99.3% nucleotide identity in the HA gene, indicating that the HA genes of the two viruses likely originated from the same source. Both BP/HuN/414/10 and CP/XH/420/10 were most closely related to the A/wild duck/Mongolia/1-241/2008 (H7N9) strain, with 97.9 and 98.3% nucleotide identities, respectively (Table 2). However, a more distant genetic relationship with the A/Shanghai/13/2013 (H7N9) strain was observed, with 91.6 and 91.4% nucleotide identities, respectively. As shown in the NA phylogenetic tree (Figure 1B), both H7N1 isolates belong to the Eurasian lineage and share the greatest sequence homology (99.2 and 99.4%) with the A/wild bird/Korea/A13/2010 (H10N1) strain (Table 2). The highest nucleotide sequence identities relative to the internal gene fragments of the two isolated H7N1 AIVs and the phylogenetic trees of the two isolated H7N1 influenza viruses' internal genes are shown in Table 2, Figures 1C–H. Remarkably, the data showed that the constellation of the internal genome segments of the two H7N1 viruses were substantially different. While the PB1, PB2, PA, NP, M, and NS genes of CP/XH/420/10 originated from H9N2, H4N8, H7N7, H7N7, H4N6, and H4N6 viruses, respectively, the PB1, PB2, PA, NP, M, and NS genes of BP/HuN/414/10 originated from H9N2, H4N8, H7N7, H5N9, H2N1, and H1N1 viruses, respectively. These results indicated that the two H7N1 strains have arisen through reassortment with different progenitor viruses.

**Table 2:** **The highest nucleotide identity of the whole genomes of two H7N1 influenza viruses**.

**Gene segment**	**BP/HuN/414/10(H7N1)**			**CP/XH/420/10(H7N1)**		
	**Closest viruses**	**Nucleotide identity (%)**	**Accession No**.	**Closest viruses**	**Nucleotide identity (%)**	**Accession No**.
HA	A/wild duck/Mongolia/1-241/2008(H7N9)	99	JN029686.1	A/wild duck/Mongolia/1-241/2008(H7N9)	98.9	JN029686.1
NA	A/wild bird/Korea/A13/2010(H10N1)	99.2	JN817546.1	A/wild bird/Korea/A13/2010(H10N1)	99.4	JN817546.1
PB2	A/duck/Nanjing/1102/2010(H4N8)	96.2	KC683700.1	A/duck/Nanjing/1102/2010(H4N8)	99.5	KC683700.1
PB1	A/avian/Egypt/920431/2006(H9N2)	97.8	GU050308.1	A/avian/Egypt/920431/2006(H9N2)	98.6	GU050308.1
PA	A/wild bird/Korea/A331/2009(H7N7)	99.1	JN244165.1	A/wild bird/Korea/A331/2009(H7N7)	99.4	JN244165.1
M	A/mallard/Sweden/113/02(H2N1)	99.7	CY122060.1	A/wild duck/Korea/CSM4-28/2010(H4N6)	99.5	JX454691.1
NP	A/chicken/Italy/9097/1997(H5N9)	98	EF597341.1	A/mallard/Korea/NHG187/2008(H7N7)	99.1	KC609905.1
NS	A/mallard/Bavaria/185-26/2008(H1N1)	98.8	HQ259236.1	A/mallard/ZhaLong/88/2004(H4N6)	98.7	FJ349250.1

### Chicken and duck experiments

**Figure 2:**
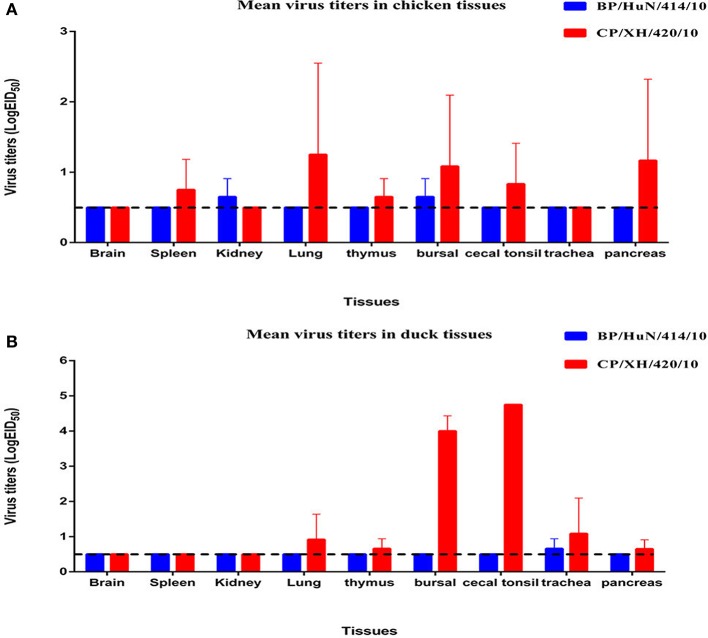
**Viral titers of the two isolated H7N1 viruses in the organs of chickens (A) and ducks (B) at 3 dpi**. Viral titers are shown as the mean ± standard deviation. The dashed line indicates the lower limit of viral detection.

Throughout the experiment, no chickens or ducks showed any clinical symptoms or mortality. We did not detect any BP/HuN/414/10 in the oropharyngeal or cloacal samples from the chickens or ducks after inoculation (Table 3). However, the virus replicated at the tested organs of chickens, including kidney and bursal samples, and in the trachea of ducks (Figures 2A,B). In the inoculated group, seroconversion against BP/HuN/414/10 was detected by the HI assay in all chickens at 14 and 21 dpi (Table 3). In the contact group, seroconversion against BP/HuN/414/10 was detected by the HI assay in two of the three chickens at 21 dpi. However, the replication capacity of the isolated CP/XH/420/10 (H7N1) virus in ducks and chickens showed obvious differences. In the inoculated group, the virus was detected in chicken cloacal and oropharyngeal samples at 3, 5, and 7 dpi, but in the contact group, the virus was only detected at 5 dpi in one oropharyngeal sample and at 7 dpi in two of the cloacal samples. In the inoculated group, we detected the virus in duck cloacal samples at 3, 5, and 7 dpi, but in the oropharyngeal samples, the virus was only detected at 3 and 7 dpi. In the contact group, a virus titer was detected in one oropharyngeal sample at 3 dpi and in cloacal samples at 5 and 7 dpi (Table 3). At 3 dpi, viral replication was detected in many organs of the euthanized chickens and ducks (Figures 2A,B). To understand the antibody responses after infection, serum samples were collected from each bird for detection of antibodies by HI assay at 14 and 21 dpi: 60% of the inoculated chickens were seropositive at 14 dpi, and 80% were seropositive at 21 dpi. Whereas only 1 of the 3 contact chickens was seropositive at 14 dpi and all 3 were seronegative at 21 dpi. However, seroconversion was detected in all of the remaining ducks, suggesting that the isolated H7N1 CP/XH/420/10 strain stimulates a better immune response in ducks (Table 3).

**Table 3:** **Animal experiments with two H7N1 AIVs in chickens and ducks**.

**Birds**	**Viruses**	**Infected method**	**3 dpi**		**5 dpi**		**7 dpi**		**Sera-conversion (positive/total)**	
			**OP**	**CL**	**OP**	**CL**	**OP**	**CL**	**14 dpi**	**21 dpi**
Chickens	BP/HuN/414/10	Inoculated	0/8^a^	0/8	0/5	0/5	0/5	0/5	5/5(2.19)^c^	5/5(4.15)
	(H7N1)	Contacted	0/3	0/3	0/3	0/3	0/3	0/3	0/3	2/3(1.15)
	CP/XH/420/10	Inoculated	6/8(<1)^b^	2/8(<1)	4/5(<1)	1/5(<1)	1/5(<1)	1/5(<1)	3/5(5.10)	4/5(5.67)
	(H7N1)	Contacted	0/3	0/3	1/3(<1)	0/3	0/3	2/3(<1)	1/3(4.69)	0/3
Ducks	BP/HuN/414/10	Inoculated	0/8	0/8	0/5	0/5	0/5	0/5	5/5(3)	0/5
	(H7N1)	Contacted	0/3	0/3	0/3	0/3	0/3	0/3	1/3(1.15)	0/3
	CP/XH/420/10	Inoculated	2/8(<1)	6/8(<1)	0/5	5/5(<1)	2/5(<1)	3/5(<1)	5/5(4.43)	5/5(5.51)
	(H7N1)	Contacted	1/3(<1)	0/3	0/3	3/3(<1)	0/3	2/3(<1)	3/3(2.31)	3/3(3.83)

*Abbreviations: dpi, days post-inoculation; OP, oropharyngeal swab; CL, cloacal swab*.

a *Positive birds/Total survival birds*.

b *Average viral titer of infected birds (log10EID50 ± SD)*.

c *Average antibody titer of infected birds (log2)*.

### Studies with mice

**Figure 3:**
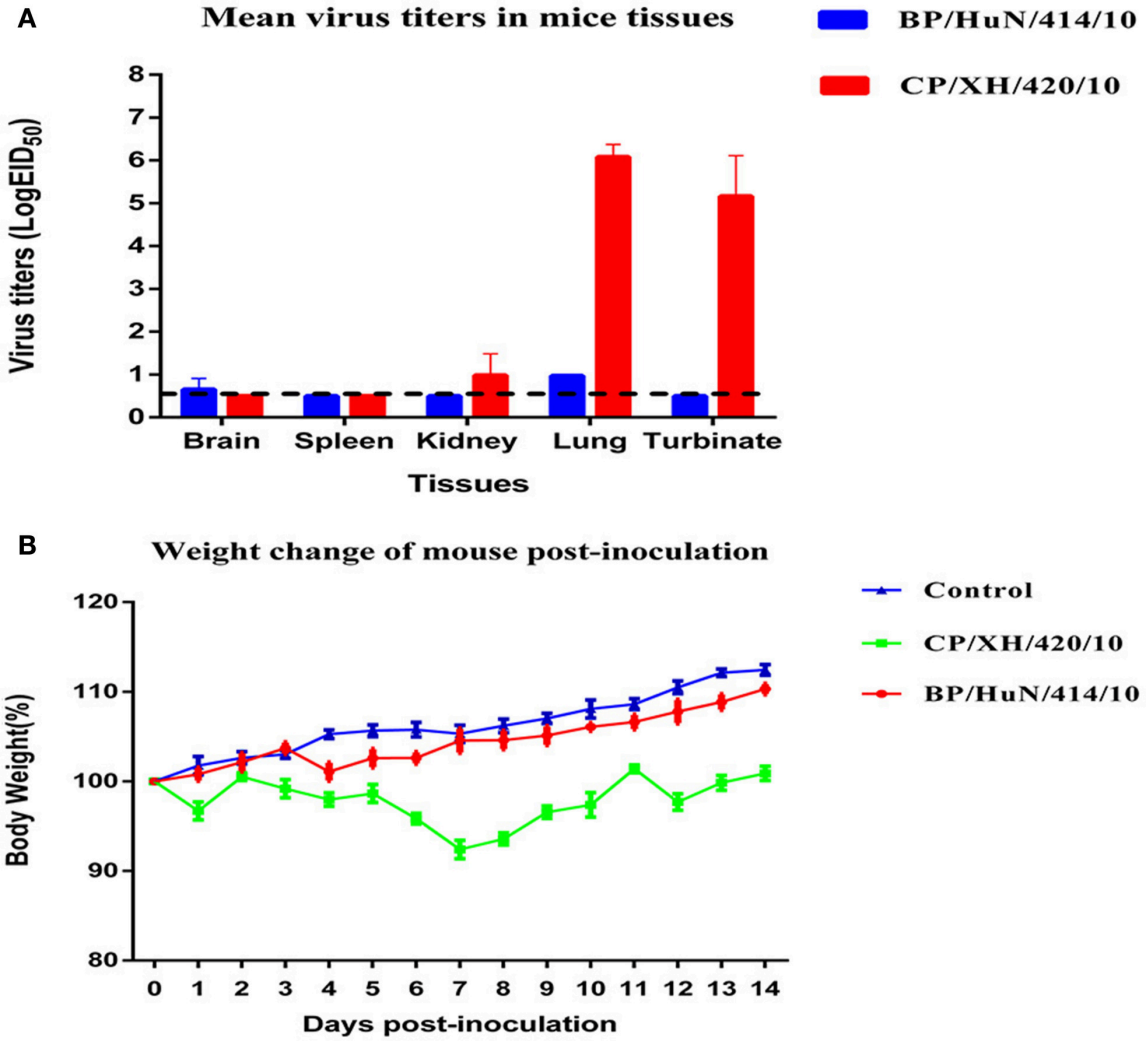
**Replication and virulence of the two isolated H7N1 viruses in mice: (A) Viral titers in the various tissues of the mice at 3 dpi**. Viral titers are shown as the mean ± standard deviation. The dashed line indicates the lower limit of viral detection. **(B)** Weight changes in mice throughout the experiment. Weight changes are shown as the mean ± standard deviation. The weight changes of the CP/XH/420/10 and control groups differed significantly (*P* < 0.05). The BP/HuN/414/10 and control groups showed no significant difference (*P* > 0.05).

**Figure 4:**
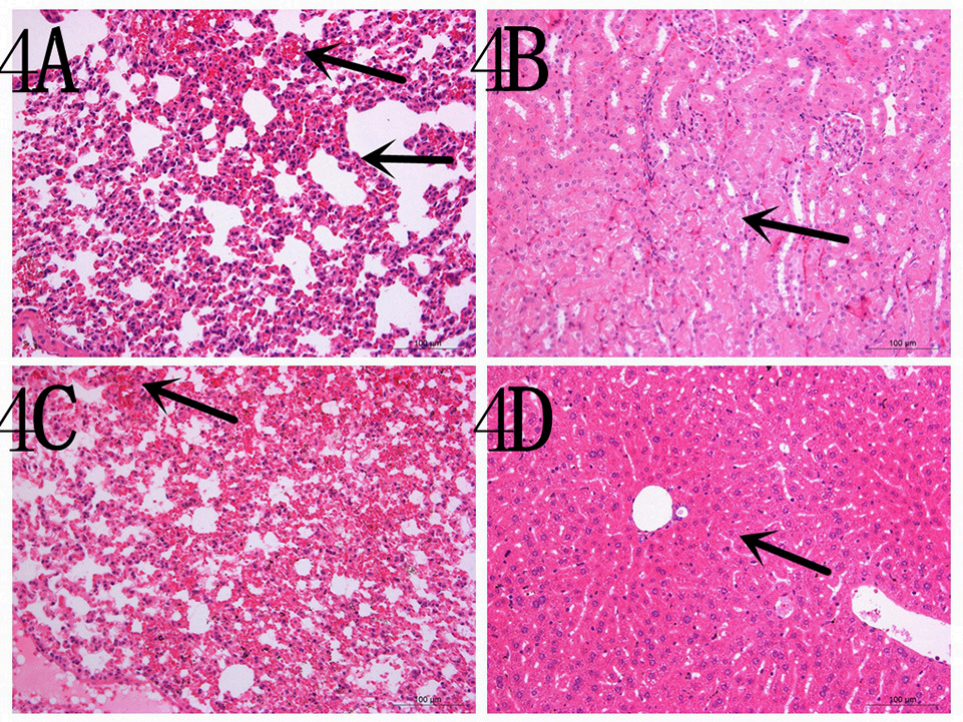
**Representative histopathological changes in the organs of the experimental mice (A) inoculated with the BP/HuN/414/10 (H7N1) virus and (B–D) with the CP/XH/420/10 (H7N1) virus**. **(A)** Pulmonary congestion and moderate broadening of the alveolar septum. **(B)** Granular degeneration of renal tubular epithelial cells. **(C)** Pulmonary congestion. **(D)** Mild granular degeneration of some liver cells.

To determine the capacity of the H7N1 AIVs to replicate and become pathogenic in mammals, we tested the two viruses in mice. The results showed that the two H7N1 isolates replicated in the lung, but the levels of the CP/XH/420/10 strain were higher than those of the BP/HuN/414/10 strain. In addition, the BP/HuN/414/10 strain replicated in the brain, and the CP/XH/420/10 strain replicated in the kidney and turbinate (Figure 3A). The difference in body weight loss at different dpi between the CP/XH/420/10 and controls groups was statistically significant (*P* < 0.05), but the difference between the BP/HuN/414/10 and controls groups was not (*P* > 0.05; Figure 3B). The pathological sections revealed pulmonary congestion and moderate broadening of the alveolar diaphragm due to the BP/HuN/414/10 (H7N1) virus infection in the mouse lung. CP/XH/420/10 (H7N1) caused pathological changes in many organs, including the lung, kidney and liver (Figure 4).

### Analysis of receptor specificity of two strains of H7N1

The chicken red blood cells exhibit α-2,3-linked and α-2,6-linked sialic acid receptors. In contrast, the sheep red blood cells exhibited only α-2,3-linked sialic acid receptors.

**Figure 5:**
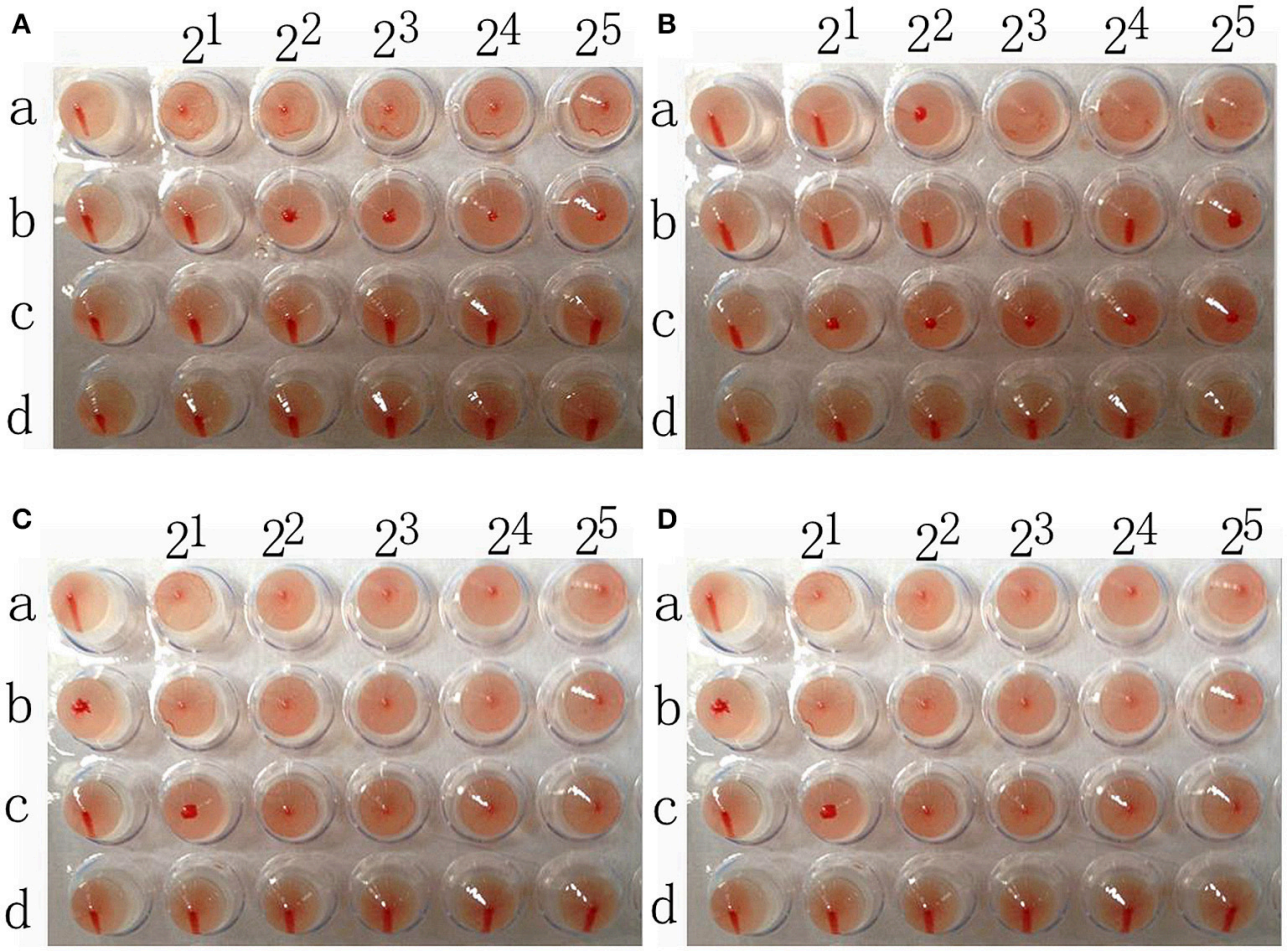
**The results of A/chicken/Jilin/HU/02 (H5N1) (A), A/Jilin/31/2005 (H1N1) (B), A/Baer's Pochard/Hunan/414/2010 (H7N1) (C), and A/Common Pochard/Xianghai/420/2010 (H7N1) (D) agglutination of different red blood cells**. a: Chicken red blood cells (with α-2,3-linked sialic acid receptors and α-2,6-linked sialic acid receptors). b: Sheep red blood cells (with only α-2,3-linked sialic acid receptors). c: Chicken red blood cells treated with α-2,3-sialidase (with only α-2,6-linked sialic acid receptors). d: Chicken red blood cells treated with VCNA (no receptors).

The results showed that the A/Chicken/Jilin/HU/02 (H5N1) strain agglutinated chicken and sheep red blood cells but could not agglutinate chicken red blood cells treated with α-2,3-sialidase that have only α-2,6-linked sialic acid receptors indicating the avian receptor specificity. The A/Jilin/31/2005 (H1N1) strain agglutinated chicken red blood cells and chicken red blood cells treated with α-2,3-sialidase that have only α-2,6-linked sialic acid receptors but could not agglutinate sheep red blood cells that only have α-2,3-linked sialic acid receptors indicating the human receptor specificity. Furthermore, the two H7N1 AIVs agglutinated chicken red blood cells, chicken red blood cells treated with α-2,3-sialidase and sheep red blood cells, showing that they possess both avian and human receptor specificity (Figure 5).

## Discussion

LPAI H7 viruses, such as the H7N9 virus, were first reported in China in February and March 2013 and pose a serious threat to human health (Gao et al., 2013). The two isolated H7N1 viruses were used to study the phylogenetic and pathogenic characteristics of H7 LPAI's in this work. The two isolated H7N1 viruses both contain the motif PELPKGR↓GLFGAI at the cleavage site between HA1 and HA2, and the A/Shanghai/13/2013 (H7N9) strain carries the motif PEIPKGR↓GLFGAI at the cleavage site between HA1 and HA2 (Table 1); however, as none of these viruses shows serial basic amino acids in the motif, they meet the criteria for low pathogenicity (Steinhauer, 1999). In the analysis of the key amino acid sites, Q226L and G228S mutations were not found in the HA protein (Table 1), showing that the two H7N1 viruses retain the ability to preferentially bind to α-2,3-linked sialic acid receptors, which is a primary characteristic of AIVs (Yamada et al., 2006). Q226L and G228S mutations in the HA protein of A/Shanghai/13/2013 (H7N9) were not found. However, A/Shanghai/13/2013 (H7N9) and the two isolated H7N1 viruses presented mutations at positions S138A and T160A of the HA protein, which may favor mammalian adaptation and increase the affinity for α-2,6-linked sialic acid receptors (Ha et al., 2001; Wan and Perez, 2007; Fan et al., 2015). The two isolated viruses had no mutations at positions E627K and D701N of the PB2 protein, whereas A/Shanghai/13/2013 (H7N9) exhibits a mutation at position E627K, which plays an important role in the adaptation of AIVs to mammals (Katz et al., 2000; Li et al., 2005). No mutation at position S31N in the M2 protein was found, indicating that the two H7N1 viruses are sensitive to amantadine inhibitors (Lee et al., 2008), but a mutation was detected in the A/Shanghai/13/2013 (H7N9) strain. In addition, the two isolated H7N1 viruses and the A/Shanghai/13/2013 (H7N9) strain present mutations at position P42S of the NS1 protein, which may increase virulence in mice (Jiao et al., 2008). These results suggest that the two H7N1 AIVs can infect mammals and may have potential to infect humans.

Phylogenetic analysis based on the complete nucleotide sequences of the HA and NA genes showed that the two isolated LPAI H7N1 viruses cluster in the Eurasian branch. Homology analysis showed 98.4% homology between the entire genomes of the two H7N1 virus strains and the homologies of the eight gene segments of the two virus strains are 96.2% (PB2), 98.7% (PB1), 99.5% (PA), 99.3% (H7), 97.1% (NP), 99.3% (N1), 98.3% (M), and 99.8% (NS), indicating that both viruses most likely obtained their HA, NA, PA, and NS genes from a recent common ancestor, whereas they most likely obtained their other internal genes through reassortment with other influenza viruses. Given that the two strains originated from different places, with one from Hunan and the other from Jilin, the migration of birds may have played a role in promoting the spread of the virus over long distances.

No virus was detected in the oropharyngeal or cloacal samples of chickens or ducks after inoculation with the BP/HuN/414/10 virus. Chickens and ducks in the contact group also did not exhibit any viral titers, but seroconversion was detected in this group, suggesting that the virus can be transmitted within species by contact (Table 3). We also found an unusual phenomenon in that all 5 inoculated ducks were seropositive at 14 dpi, but no ducks were seropositive at 21 dpi. Similarly, it was odd that 1 of the 3 contact ducks exposed to the same virus (BP/HuN/414/10) was seropositive at 14 dpi, but none of the contact ducks were seropositive at 21 dpi (Table 3). These results show that the immunogenicity of the BP/HuN/414/10 virus is weak, and it is not able to stimulate the duck body to produce an effective humoral immune response. Therefore, the duration of the antibodies in the duck body is less than 21 days. The LPAI BP/HuN/414/10 virus replicated at the chicken kidney and bursal tissue as well as the duck trachea indicating that the virus can infect chickens and ducks. However, in contrast with BP/HuN/414/10, the replication and infection ability of the isolated CP/XH/420/10 (H7N1) virus in chickens and ducks showed obvious differences. Unlike BP/HuN/414/10, the virus CP/XH/420/10 (H7N1) was detected in both the oropharyngeal and cloacal samples of inoculated and contact chickens and ducks (Table 3), indicating that the isolated CP/XH/420/10 strain can be transmitted between species by contact. The HI assay showed that 60% of the chickens inoculated with CP/XH/420/10 were seropositive at 14 dpi, and 80% were seropositive at 21 dpi. However, only one of the 3 contacted chickens exposed to CP/XH/420/10 was seropositive at day 14, and none were seropositive at day 21 (Table 3). This difference in seropositivity between inoculated and contact chickens may be related to the amount of virus each chicken received as presumably the dose received by chickens in the directly inoculated group was higher than those in the contact group. In addition, the virus CP/XH/420/10 can replicate efficiently in multiple organs of chickens and ducks (Figures 2A,B), particularly in duck bursa and tonsils showing stronger replication capacity than BP/HuN/414/10. Ducks showed higher virus titer in bursa and tonsils which were avian immune organs is possible related to the immune system capture, aggregation and antigen clearing. Although, the pattern of infection in the two strains differed in some ways, both strains could infect chickens and ducks. Live poultry markets appear to be an important source of human infection (Chen et al., 2013; Li et al., 2014), and according to the literature, H7 subtypes of LPAI viruses have been reported to evolve into a highly pathogenic avian influenza virus in poultry (Horimoto and Kawaoka, 1995; Banks et al., 2001; Lee et al., 2005). Therefore, it is necessary to carry out AIV surveillance in live poultry markets.

In our study, the average weight change of mice inoculated with the two strains of H7N1 was slightly lower than that of the control group, suggesting that the two strains exhibit low pathogenicity in mice. The virulence of AIV in mice is determined by many amino acid sites, including PB2, HA, NS1, PA, and M1 mutations. The two isolated H7N1 viruses contained HA (138 and 160) and NS1 (42) mutations (Table 1). The virulence of CP/XH/420/10 was higher than that of BP/HuN/414/10, suggesting that some unknown factors may play important roles in the pathogenicity in mice (Figure 3B). According to the results of the animal experiments as a whole, including the infection of chickens, ducks and mice, the CP/XH/420/10 virus was more pathogenic than the BP/HuN/414/10 virus. We speculate that this phenomenon may be related to the differences in amino acid sites between the two viruses (Table 4), but we do not know which amino acid site would cause this difference. Therefore, these different amino acid sites will be studied by reverse genetics in our future work.

**Table 4:** **Analysis of different amino acid sites in BP/HuN/414/10 (H7N1) and CP/XH/420/10 (H7N1)**.

**Virus**	**HA (H7 numbering)**			**NA**		**PB2**			**NP**	**PB1**		**M1**
	**95**	**228**	**319**	**43**	**355**	**292**	**340**	**513**	**105**	**428**	**596**	**92**
BP/HuN/414/10(H7N1)	I	A	K	R	D	V	R	F	M	H	S	N
CP/XH/420/10(H7N1)	V	T	R	Q	N	I	K	L	V	Q	P	K

The HA protein's specificity for binding surface receptors in the host cell is a prerequisite for viral infection of the host. Human influenza viruses preferentially bind to α-2,6-linked sialic acid receptors, and avian influenza viruses preferentially bind to α-2,3-linked sialic acid receptors. In this study, the two isolated H7N1 viruses were able to bind to both α-2,3-linked sialic acid receptors and α-2,6-linked sialic acid receptors. This conclusion is consistent with the results that showed that BP/HuN/414/10 and CP/XH/420/10 could infect chickens, ducks and mice.

In conclusion, the results indicated that the two isolated H7N1 strains could infect mammals. Moreover, H7N1 AIVs are circulating widely in many areas, including North America (Panigrahy et al., 1995), Europe (Brown, 2010; Gonzales et al., 2011), southern China (Peng et al., 2014), New Zealand and Australia (Bulach et al., 2010). A previous study also found that an H7N1 AIV with no history of human infection could obtain capacity of airborne transmission among ferrets introduced by serial passage test (Sutton et al., 2014). Therefore, it is necessary to strengthen AIV surveillance in wild birds and poultry.

## Author contributions

HJ contribute on experiment, writing article. DW contribute on isolation and identification of virus. JS collected the samples and performed the experiment. HJ, DW, and JS are co-senior authors. YH, HC, YG, and YL guided the experiment and are the corresponding authors. YC contribute on sequencing. GC collected the samples. XZ, JZ, and XL helped to perform the experiments.

### Conflict of interest statement

The authors declare that the research was conducted in the absence of any commercial or financial relationships that could be construed as a potential conflict of interest.
