# Supplementary material for: Pathogenesis and Phylogenetic Analyses of Two Avian Influenza H7N1 Viruses Isolated from Wild Birds
Source: Front Microbiol. 2016 Jul 7;7:1066. doi: 10.3389/fmicb.2016.01066 (PMC4935687; doi:10.3389/fmicb.2016.01066)
Supplement: Supplementary file 2 [file Table2.DOC]

| H7--1 | GCAAAAGCAGGGGATACAAA |
| --- | --- |
| H7--2 | AGTTTTTTCCAAACTTATATAC |
| N1--1 | AGATTAAAATGAATCCAAATCAG |
| N1--2 | TTTGAACAAACTACTTGTCAATGG |
